# Protective effects of biochanin A on articular cartilage: *in vitro* and *in vivo* studies

**DOI:** 10.1186/1472-6882-14-444

**Published:** 2014-11-15

**Authors:** Ding-Qian Wu, Hui-ming Zhong, Qian-hai Ding, Li Ba

**Affiliations:** Department of Emergency Medicine, The Second Affiliated Hospital of School of Medicine, Zhejiang University, Jie Fang Road 88#, Hangzhou, 310009 People’s Republic of China; Research Institute of Emergency Medicine, Jie Fang Road 88#, Hangzhou, 310009 People’s Republic of China; Department of Orthopedic Surgery, The Second Affiliated Hospital of School of Medicine, Zhejiang University, Jie Fang Road 88#, Hangzhou, 310009 People’s Republic of China

**Keywords:** Biochanin A, Osteoarthritis, Chondroprotection, Interleukin-1beta, Matrix metalloproteinases, Anterior cruciate ligament transection (ACLT)

## Abstract

**Background:**

Increased production of matrix metalloproteinases (MMPs) is closely related to the progression of osteoarthritis (OA). The present study was performed to investigate the potential value of biochanin A in inhibition of MMP expression in both rabbit chondrocytes and an animal model of OA.

**Methods:**

MTT assay was performed to assess chondrocyte survival in monolayers. The mRNA and protein expression of MMPs (including MMP-1, MMP-3, and MMP-13) and tissue inhibitor of metalloproteinase-1 (TIMP-1) in interleukin-1 < beta > (IL-1β)-induced rabbit chondrocytes were determined by quantitative real-time PCR and enzyme-linked immunosorbent assay (ELISA), respectively. The involvement of the NF-kappaB (NF-κB) pathway activated by IL-1β was determined by western blotting. The *in vivo* effects of biochanin A were evaluated by intra-articular injection in an experimental OA rabbit model induced by anterior cruciate ligament transection (ACLT).

**Results:**

Biochanin A downregulated the expression of MMPs and upregulated TIMP-1 at both the mRNA and protein levels in IL-1β-induced chondrocytes in a dose-dependent manner. In addition, IL-1β-induced activation of NF-κB was attenuated by biochanin A, as determined by western blotting. Moreover, biochanin A decreased cartilage degradation as determined by both morphological and histological analyses *in vivo*.

**Conclusions:**

Taken together, these findings suggest that biochanin A may be a useful agent in the treatment and prevention of OA.

## Background

Osteoarthritis (OA) is a multifactorial degenerative joint disorder characterized by the progressive breakdown of cartilage extracellular matrix. OA affects approximately 12% of the aging population in western countries, while a quarter of people aged over 55 have an episode of persistent knee pain [1]. There is still no effective treatment to block the progression of OA. Other than surgical therapies, treatment for OA has generally been aimed at alleviating the major complaints, such as swelling, pain, and muscle tightness.

Homeostasis of the cartilage is maintained by the balance between the anabolic and catabolic activities of the articular chondrocytes [2]. The aggregating proteoglycan along with type II collagen provides robust mechanical properties to cartilage in a healthy joint. Increased type II collagen breakdown by collagenases and aggrecan cleavage by aggrecanases ultimately lead to joint cartilage destruction and exposure of the underlying bone [3]. The matrix metalloproteinases (MMPs) are thought to be the key matrix degradation enzymes due to their ability to cleave most components of the extracellular matrix [4]. In addition, the imbalance between MMPs and tissue inhibitors of metalloproteinases (TIMPs) plays a pathophysiological role in the progression of OA [5]. Inflammatory cytokines, such as interleukin-1 beta (IL-1β), tumor necrosis factor-alpha (TNF-α), and IL-6, have been detected in the synovial fluid of OA patients [6]. For example, IL-1β has been reported to contribute to OA progression by inducing MMP expression as well as other catabolic factors [7].

Currently, there is growing interest in compounds extracted from plants that possess significant value in the treatment of OA for their potent antiarthritic effects and minimal side effects. Biochanin A, an isoflavone found in red clover, has been shown to possess anticancer, antiallergic, and anti-inflammatory effects [8]. A recent study indicated that biochanin A exerted antiproliferative and anti-inflammatory effects through the inhibition of iNOS expression, p38-MAPK and ATF-2 phosphorylation, and blocking of NF-κB nuclear translocation [9]. A previous study indicated that biochanin A inhibited tumor invasion in human glioblastoma (U87MG) cells by suppressing the enzymatic activities of MMP-2 and MMP-9 [10]. As MMPs are regarded as major factors in the pathophysiology of OA, this close link between biochanin A and MMPs prompted us to explore whether biochanin A could have a protective effect in OA by regulating MMPs. The present study was performed to determine the effects of biochanin A treatment on osteoarthritis *in vitro* and *in vivo*.

## Methods

### Reagents

Biochanin A and 3-(4, 5-Dimethyl-2-thiazolyl)-2, 5-diphenyl-2H-tetrazolium bromide (MTT) were obtained from Sigma-Aldrich (St Louis, MO, USA). Recombinatnt interleukin-1beta was purchased from Peprotech Group (USA). Dul-becco‘s Modified Eagle’s Medium (DMEM), penicillin and streptomycin, fetal bovine serum (FBS), 0.05% trypsin and collagenase II were obtained from GibcoBRL (Grand Island, NY, USA). Biochanin A was dissolved in dimethyl sulphoxide (DMSO) and filtered prior to use.

### Isolation and culture of chondrocytes

The study was approved by the Institutional Animal Care and Use Committee of Zhejiang University (Hangzhou, China) and conducted in accordance with the Guide for the Care and Use of Laboratory Animals as adopted and promulgated by the United States National Institutes of Health. Normal articular cartilage was collected from the tibial plateau and femoral condyle of 4-week-old New Zealand rabbits. Cartilage was cut into thin slices and digested with 0.4% collagenase II in DMEM at 37°C for 4 h. The released cells were centrifuged, resuspended, and cultured in 75-cm^2^ culture flasks in complete DMEM supplemented with 10% FBS and antibiotics (100 units/mL penicillin, 100 μg/mL streptomycin). Confluent primary chondrocytes were passaged at a ratio of 1:3. Chondrocytes from passages two to three were used for our experiments.

### Assessment of cell viability

The cytotoxicity of biochanin A to rabbit chondrocytes was evaluated in the presence of increasing concentrations of biochanin A (0, 5, 25, 50 μM) by using the MTT assay according to the instructions of the manufacturer as previously described [11]. Briefly, rabbit chondrocytes were seeded onto 96-well plates at a density of 5 × 10^3^/well. Various concentrations of biochanin A (5, 25, 50 μM) was added to cultured wells and incubated for 24 h. At the indicated time, 20 μL of MTT solution (5 mg/ml in phosphate-buffered saline) was added to each well and incubated for another 4 h at 37°C. After removement of supernatant, 150 μL of DMSO was added to each well and absorbance at 570 nm was measured using a microplate reader (Bio-Rad, Hercules, CA, USA).

### Quantitative real-time polymerase chain reaction (PCR)

Serum-starved chondrocytes were pretreated with various concentrations of biochanin A for 2 h followed by co-incubation with IL-1β for 24 h. At the indicated time, the monolayer chondrocytes were harvested for extraction of total RNA by using Trizol Reagent (Invitrogen, Carlsbad, CA), while the culture supernatant was collected for storing at −80°C until use. First complementary DNA (cDNA) was synthesized by using a primescript –RT reagent kit (TaKaRa Biotechnology Co, Ltd, Japan). Quantitative real-time PCR was performed in 20 μL reactions by the following procedures: followed by 40 cycles of 95°C for 15 seconds and 60°C for 32 seconds. The following primers were used: for MMP-1, 5’-atgctgaaaccctgaagatgat-3’ (forward) and 5’- ccttggagactttggtgaatgt-3’ (reverse); for MMP-3, 5’- cgttcctgatgttggtcactt -3’ (forward) and 5’- tcagcctctccttcatacttcc -3’ (reverse); for MMP-13, 5’-cccctcctcaacagtaacgag-3’ (forward) and 5’- agtttgcctgtcacctctaagc-3’ (reverse); for TIMP-1, 5’-gggctccagaagtcaatcatac-3’ (forward) and 5’-tacccgcagacactttccat-3’ (reverse); for 18S, 5’-cgtagttccgaccataaacgat-3’ (forward), 5’- aatctgtcaatcctgtccgtgt-3’ (reverse). Expression of 18S was used as endogenous control. Amplification and quantification of relative expression levels of MMP-1, MMP-3, MMP-13 and TIMP-1 was determined by ΔΔCT method [12].

### ELISA

The effect of IL-1β and/or biochanin A on the level of MMP-1, MMP-3, MMP-13 and TIMP-1 secreted by rabbit chondrocytes in the culture supernatant mentioned above was furthered detected by using commercially available ELISA kit (R&D Systems, Inc., Minneapolis, MN,USA) and all the assays were performed in accordance with the manufacturer’s instructions.

### Western blot analysis

Confluent rabbit chondrocytes were starved of serum overnight and pretreated with various concentrations of biochanin A for 2 h, followed by coincubation with IL-1β for 24 h. Cells were washed with ice-cold PBS and lysed with lysis buffer. The protein samples were separated by 12% sodium dodecyl sulfate-polyacrylamide gel electrophoresis (SDS-PAGE) and transferred onto PVDF membranes. The membranes were blocked with 5% non-fat milk dissolved in TBST buffer and probed with primary antibodies against MMP-1, MMP-3, MMP-13, TIMP-1 (Proteintech, Chicago, IL), IκB-α, NF-κB p65, and β-actin (Cell Signaling Technology, Danvers, MA) at 4°C overnight. The membranes were washed with TBST and incubated with appropriate HRP-labeled secondary antibodies for 1 h at room temperature. Detection was carried out using enhanced chemiluminescent (ECL) substrate and exposure to Kodak X-Omat film (Kodak, Rochester, NY) according to the manufacturer’s protocols.

### Animal studies

Rabbits were obtained from the Animal Center of Zhejiang University and the procedures were approved by the Institutional Animal Care and Use Committee of Zhejiang University, Hangzhou, China. Twelve mature New Zealand rabbits underwent bilateral anterior cruciate ligament transection (ACLT) and divided randomly into the low-concentration (A) and high-concentration (B) biochanin A treatment groups. Four weeks after ACLT surgery, rabbits in groups A and B received an intra-articular injection of 0.3 mL of 5 and 25 μM biochanin A, respectively, into the left knee and 0.3 mL of DMSO (vehicle) into the right knee. Weekly administration continued for 5 weeks. All rabbits were sacrificed after 9 weeks of ACLT surgery. Four rabbits were used as a normal control group.

### Histological assessment

Collected specimens of femoral condyle were fixed in 10% neutral-buffered formalin for 72 h. After dehydration through an alcohol series, specimens were embedded in paraffin, cut into sections 7 μm thick, and stained with Safranin-O-fast green. The degree of cartilage degradation was scored according to the Mankin system by two independent researchers in a blinded manner [13].

### Evaluation of cartilage gene expression by quantitative real-time PCR

Collected cartilage was frozen in liquid nitrogen prior to use. The mRNA levels of MMP-1, MMP-3, MMP-13, and TIMP-1 in all cartilage samples were assessed by quantitative real-time PCR as described above.

### Statistical analysis

All experiments were performed duplicated and repeated three times using independent samples. The results are expressed as means  ±  standard deviation (SD). One-way ANOVA followed by Dunnett’s analysis was used for statistical analysis. In all analyses, *P*  <  0.05 was taken to indicate statistical significance.

## Results

### Effects of biochanin A on chondrocytes viability

**Figure 1:**
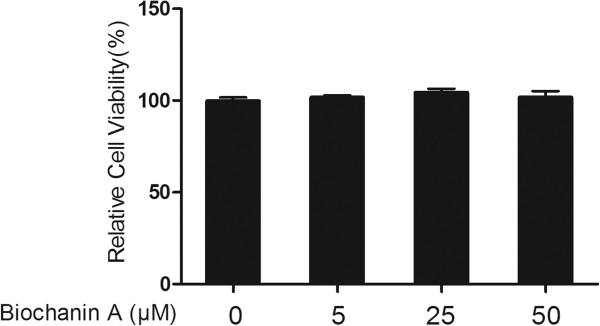
**Effects of biochanin A on chondrocytes viability.** Cells were treated with various concentrations of biochanin A for 24 h. The cytotoxicity of biochanin A toward chondrocytes was assessed by MTT assay.

MTT assay was performed to evaluate the cytotoxicity of biochanin A on rabbit chondrocytes. As shown in Figure 1, biochanin A at 5 to 50 μM exhibited no significant cytotoxicity toward rabbit chondrocytes.

### Effects of biochanin A on gene expression of MMP-1, −3, −13, and TIMP-1 induced by IL-1β in chondrocytes

**Figure 2:**
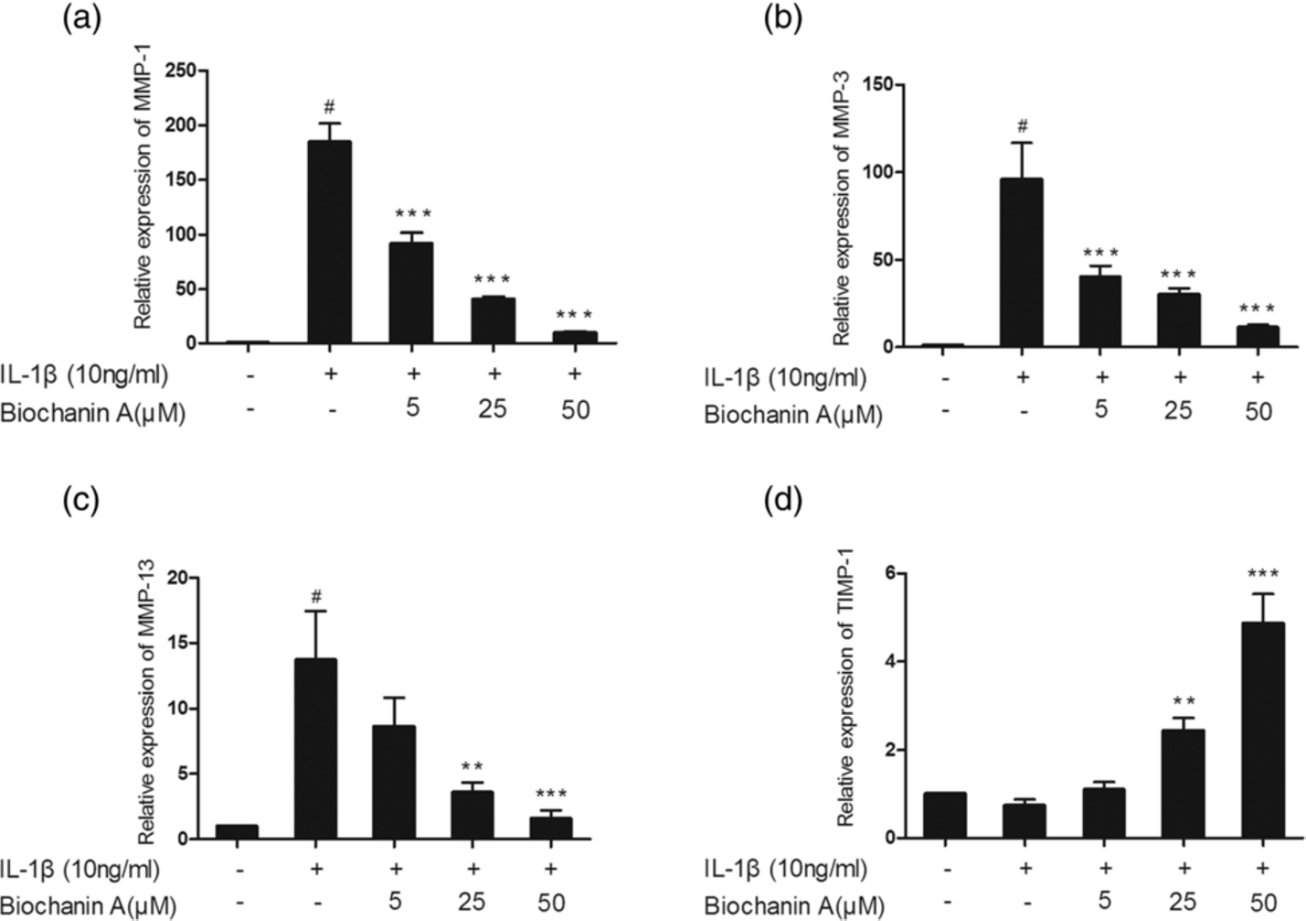
**Relative gene expression of MMP-1, −3, −13 and TIMP-1 in IL-1β -induced chondrocytes.** Cells were pre-treated with different concentrations of biochanin A for 2 h, followed by co-incubation with IL-1β (10 ng/ml) for 24 h. Data were expressed as means ± standard deviation (SD). *P < 0.05, **P < 0.01, ***P < 0.001, compared with cells stimulated with IL-1β alone; # P < 0.01, compared with normal chondrocytes. **(a)**, **(b)**, **(c)**, **(d)** represented as the relative gene expression of MMP-1,MMP-3,MMP-13 and TIMP-1 respectively.

Quantitative real-time PCR was performed to assess the mRNA levels of MMP-1, −3, −13, and TIMP-1 in chondrocyte monocultures. As shown in Figure 2, IL-1β enhanced the expression of MMP-1, −3, and −13, and decreased the expression of TIMP-1. In chondrocytes pretreated with biochanin A, the upregulated MMP-1, −3, and −13 expression was markedly inhibited and the downregulated TIMP-1 expression was enhanced.

### Effects of biochanin A on protein expression of MMP-1, −3, −13, and TIMP-1

**Figure 3:**
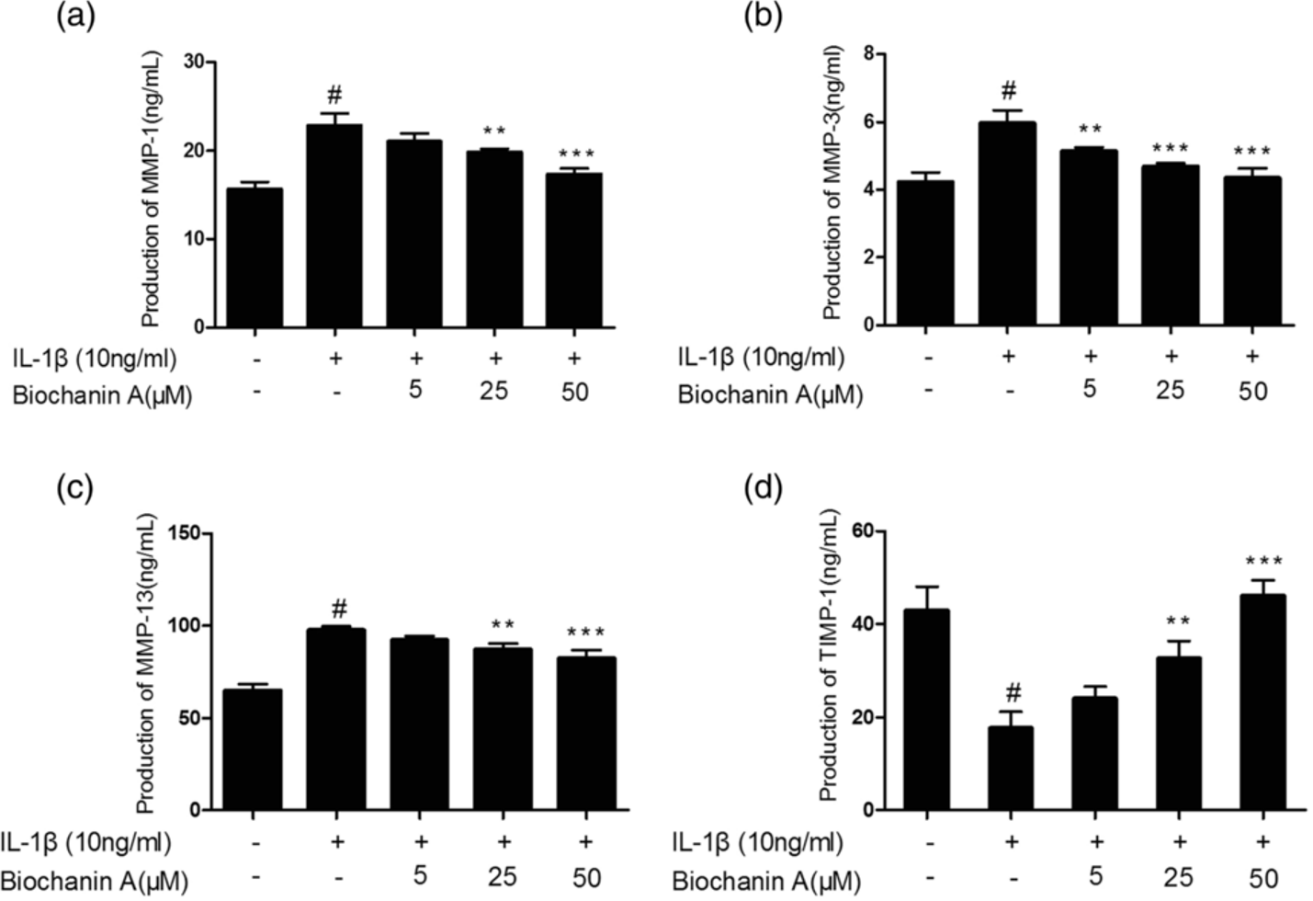
**Production of MMP-1, −3, −13, and TIMP-1 in cultured supernatant.** Production of MMP-1, −3, −13, and TIMP-1 was analyzed by enzyme-linked immunosorbent assay (ELISA). Data were expressed as means ± standard deviation (SD). *P < 0.05, **P < 0.01, ***P < 0.001, compared with cells stimulated with IL-1β alone; #P < 0.01, compared with normal chondrocytes. **(a)**, **(b)**, **(c)**, **(d)** represented as the production of MMP-1,MMP-3,MMP-13 and TIMP-1 respectively.

**Figure 4:**
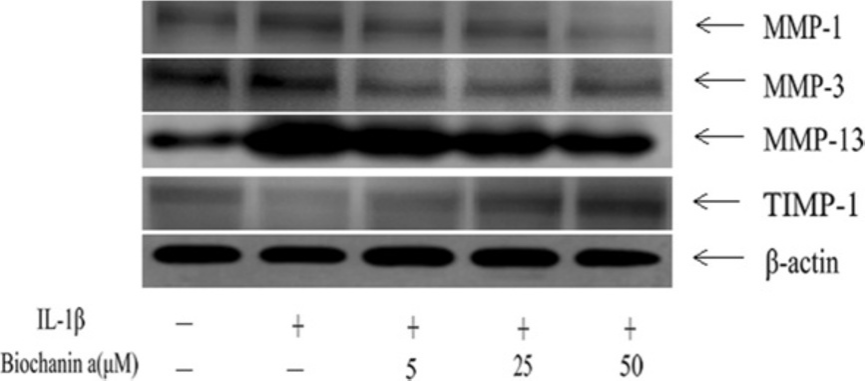
**Effects of biochanin A on protein expression of MMP-1,-3, −13, and TIMP-1 in interleukin-1β (IL-1β)–induced chondrocytes.** Chondrocytes were incubated for 24 hours with IL-1β or a combination of biochanin A and IL-1β. Protein levels of MMP-1, −3, −13, and TIMP-1 were determined by Western blotting. β-actin was used as a loading control in the Western blotting.

**Figure 5:**
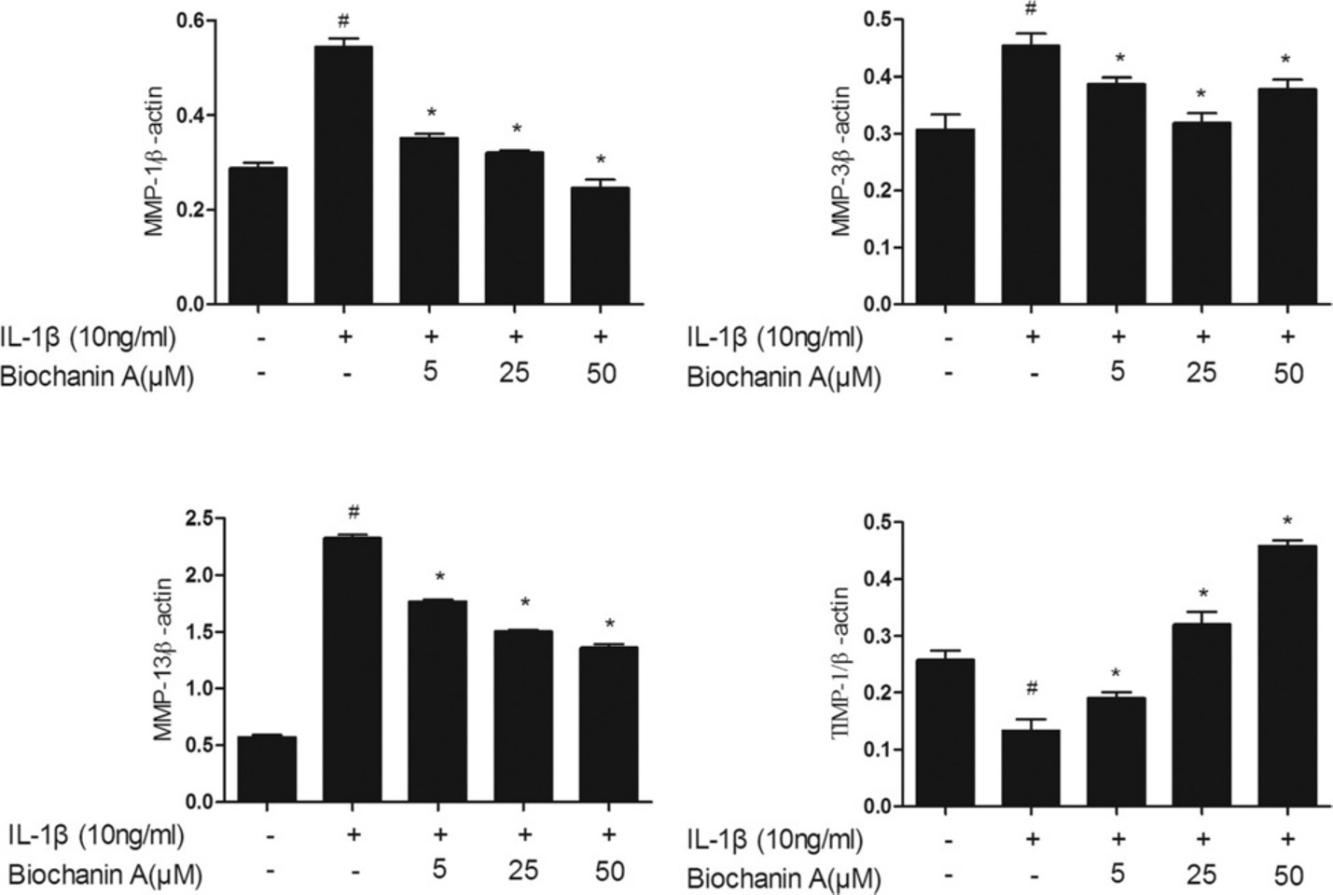
**Effects of biochanin A on protein expression of MMP-1,-3, −13, and TIMP-1 in interleukin-1β (IL-1β)–induced chondrocytes.** Ratios obtained by normalizing the expressions of MMPs, TIMP-1 IkBa and NFkB to that of beta-actin bands. *P < 0.05, compared with cells stimulated with IL-1β alone; #P < 0.05, compared with normal chondrocytes.

To further investigate MMP and TIMP-1 protein levels in IL-1β–stimulated chondrocytes, we used enzyme-linked immunosorbent assay (ELISA) and western blotting. As shown in Figures 3, 4 and 5, MMP-1, −3, and −13 protein levels were increased after IL-1β stimulation, and this effect was markedly suppressed by biochanin A in a dose-dependent manner. Although 5 μM biochanin A showed no significant effects, a higher concentration of biochanin A markedly suppressed the production of MMP-1, −3, and −13 and promoted the production of TIMP-1, consistent with the quantitative real-time PCR results.

### Effects of biochanin A on IκB-α degradation and NF-κB activation in IL-1β-treated chondrocytes

**Figure 6:**
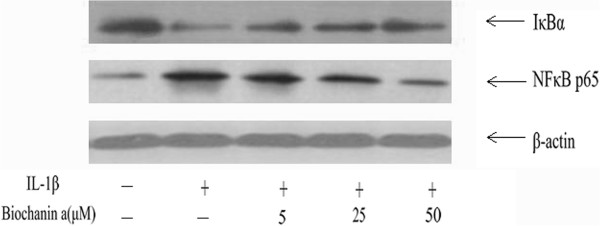
**Effects of biochanin A on IκB-α degradation and NF-κB activation in IL-1β -induced chondrocytes.** Chondrocytes were pre-treated with various concentrations of biochanin A for 2 h, followed by co-incubation with IL-1β (10 ng/ml) for 24 h, and harvested for Western blotting analysis. Biochanin A suppressed IL-1β-induced degradation of IκB-α and then attenuated IL-1β -stimulated activation of NF-κ B.

**Figure 7:**
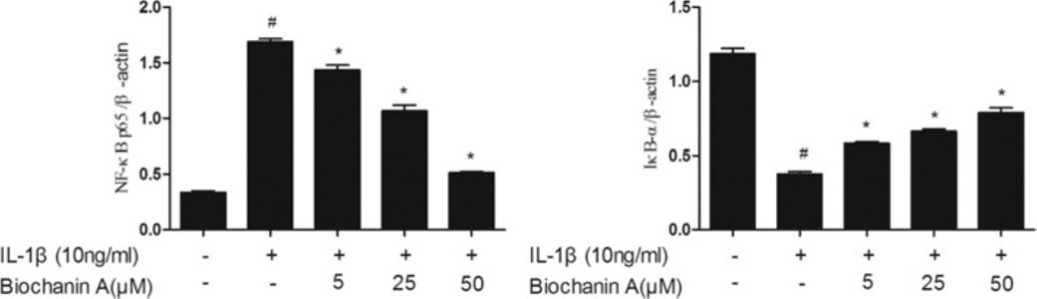
**Effects of biochanin A on IκB-α degradation and NF-κB activation in IL-1β -induced chondrocytes.** Ratios obtained by normalizing the expressions of IkB-α and NF-kB to that of beta-actin bands. *P < 0.05, compared with cells stimulated with IL-1β alone; #P < 0.05, compared with normal chondrocytes. The former and latter image represented as the relative expression of NF-κ B p65 and IkB-α respectively.

In the present study, we assayed IκB-α and NF-κB p65 to evaluate the involvement of the NF-κB pathways activated by IL-1β. As expected, biochanin A inhibited IL-1β–stimulated activation of NF-κB p65 and suppressed the IL-1β–induced degradation of IκB-α, as shown in Figures 6 and 7.

### Gross morphology and histological evaluation

**Figure 8:**
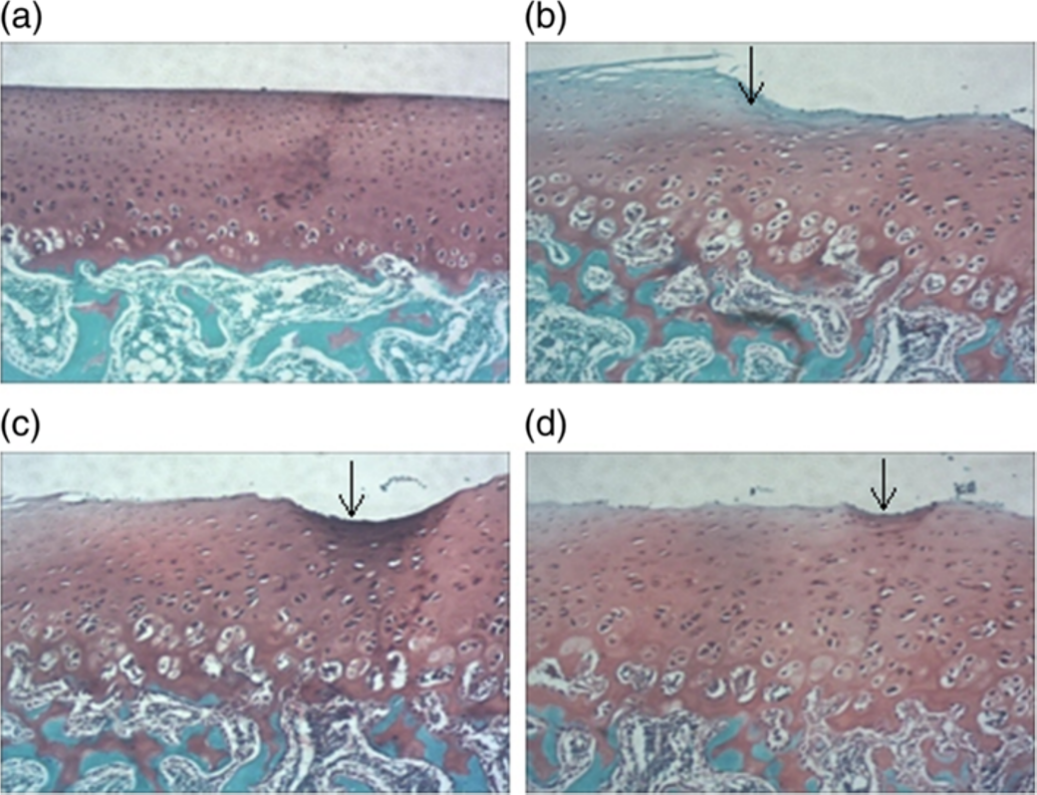
**Photographs of rabbit articular cartilage stained by Safranin-O. (a)**. Normal articular cartilage showed no reduction of Safranin staining. **(b)**. Vehicle-treated cartilage showed the loss of Safranin O staining, the arrow indicates the calcified cartilage and irregular surface. **(c)**,**(d)** biochanin A -treated cartilage showed reduction loss of Safranin O staining compared with vehicle-treated group (original magnification × 40), the arrow indicate irregular surface. **a**, **b**, **c**, **d** in Figure 8 represented as normal cartilage, vehicle-treated cartilage, biochanin A (5 μM) and biochanin A (50 μM)-treated cartilage respectively.

Our *in vivo* data revealed severe cartilage erosion in all rabbits treated by ACLT. While rabbits that received intra-articular injection of biochanin A showed less cartilage destruction compared with the vehicle-treated control group, the difference was not significant. On histological examination, cartilage specimens from the vehicle-treated group showed noticeable morphological changes, including surface irregularities, hypocellularity, and reduction of Safranin-O staining. While a significant decrease in the severity of cartilage degradation was seen in biochanin A -treated groups, particularly in the 50 μM group (Figure 8), which was consistent with histological analyses using the Mankin scoring system (Table 1).

**Table 1:** **Histological score of articular cartilage**

Femoral condyle	Vehicle	Biochanin A (5μM )	Biochanin A (50μM)
Structural changes	3.23 ± 0.31	2.76 ± 0.15	1.8 ± 0.1*
Cellular changes	2.67 ± 0.15	2.27 ± 0.25	1.87 ± 0.15*
Safranin staining	2.9 ± 0.1	2.53 ± 0.21	1.94 ± 0.15*
Total score	8.8 ± 0.53	7.57 ± 0.40*	5.6 ± 0.26*

Values are the mean ± SD. *p < 0.05 compared with vehicle group.

### Effects of biochanin A on MMP-1, −3, −13, and TIMP-1 gene expression

**Figure 9:**
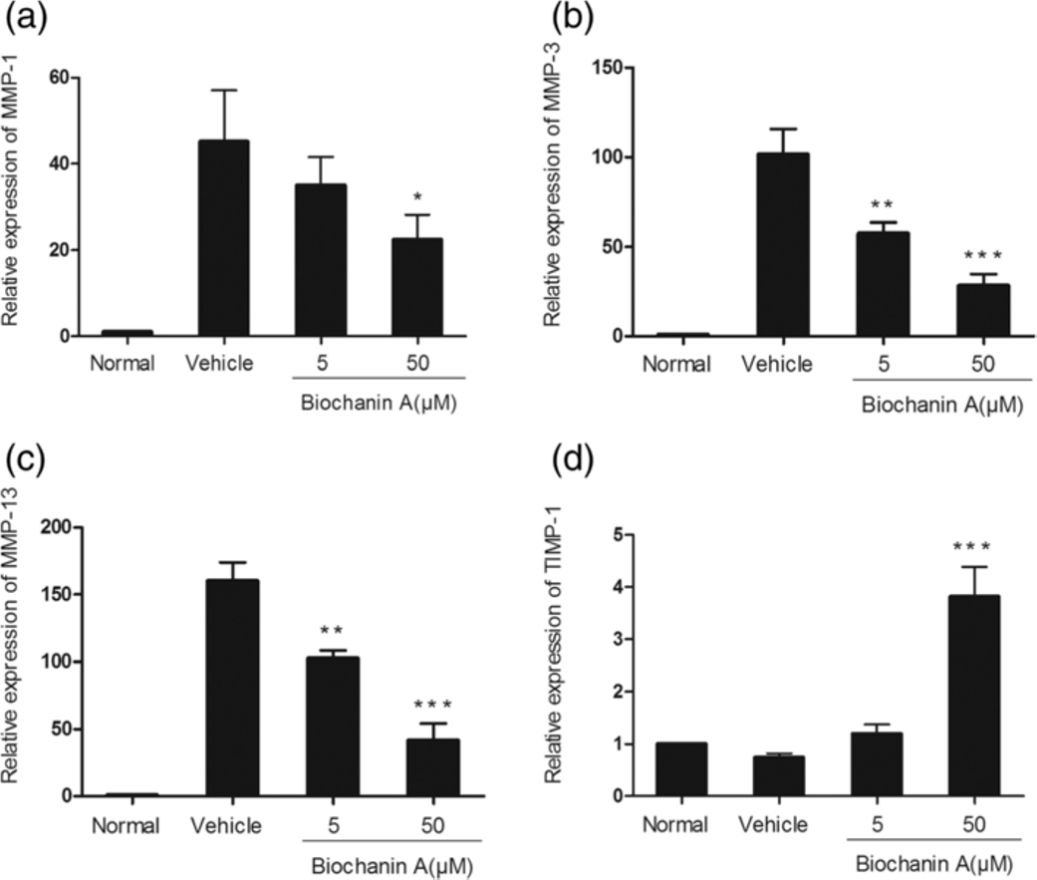
**Relative gene expression in articular cartilage.** The relative gene expression of MMP-1, −3, −13 and TIMP-1 was calculated by using quantitative real-time PCR. Data were presented as means ± standard deviation (SD). *P < 0.05, **P < 0.01, ***P < 0.001, compared with vehicle-treated cartilage. **(a)**, **(b)**, **(c)**, **(d)** represented as the relative gene expression of MMP-1,MMP-3,MMP-13 and TIMP-1 respectively.

As shown in Figure 9, in vehicle-treated cartilage of OA rabbits, the relative levels of MMP-1, −3, and −13 mRNA expression were markedly increased, while that of TIMP-1 was decreased compared to the levels in normal cartilage. However, MMP-1, −3, and −13 mRNA levels were downregulated and that of TIMP-1 was upregulated in the biochanin-A-treated group in comparison with vehicle-treated cartilage.

## Discussion

The results of the present study indicated that biochanin A potently suppressed MMP-1, −3, and −13 expression and increased that of TIMP-1 both *in vitro* and *in vivo*. In addition, our results revealed the involvement of NF-κB in MMP regulation by biochanin A.

Current therapeutic options for OA include Non-steroidal anti-inflammatory drugs [14] such as cyclooxygenase-2 inhibitors and other agents for pain relief but these drugs fail to block the progression of the disease. Apart from this, these clinical agents are associated with many risks including gastrointestinal [15], cardiovascular [16] and other adverse events. There is an increasing interest in use of compounds derived from natural plants or herbs for the treatment of OA because they have shown to be clinical efficacy with minimal side effects, as compared to routine pharmacological strategies. Biochanin A, an isoflavone derived from red clover, cabbage, and alfalfa, has been reported to exert a wide range of pharmacological effects in different experimental models. Sehdev *et al*. reported that biochanin A may be a unique natural anticancer agent that can selectively target cancer cells and inhibit multiple signaling pathways in HER-2-positive breast cancer cells [17]. Lee *et al*. suggested that biochanin A may play important physiological roles in the prevention of postmenopausal osteoporosis in osteoblastic MC3T3-E1 cells [18]. Chen *et al*. reported that biochanin A protects dopaminergic neurons against LPS-induced damage through inhibition of microglia activation and generation of proinflammatory factors [19]. In addition, biochanin A was reported to inhibit MMP-9 and MT1-MMP in human glioblastoma (U87MG) cells. However, little is known about the effects of biochanin A on osteoarthritic models or chondrocytes. Recent study showed that biochanin A prevents adipogenesis, enhances osteoblast differentiation in mesenchymal stem cells, and has beneficial regulatory effects in bone formation [20]. In view of the uncertain effects of biochanin A on osteoarthritis as well as the close link between biochanin A and MMPs, the present study was performed to explore the potential use of biochanin A *in vitro* and *in vivo*.

Osteoarthritis (OA) is characterized by the loss of the balance between anabolic and catabolic responses of stimulated chondrocytes, driven locally by a series of cytokines of which IL-1β is regarded as the major factor [21]. IL-1β is thought to be one of the most important catabolic cytokines produced by chondrocytes in OA [22]. IL-1β is known to be a potent inducer of enzymes, prostanoids, nitric oxide, and free radicals [23]. Moreover, IL-1β can suppress the synthesis and production of type II collagen and proteoglycans [24, 25]. Upregulation of IL-1β in OA cartilage tissue has been reported; indeed, increased levels of IL-1β have been detected in the synovial fluid of patients with OA [26, 27]. Our data confirmed that IL-1β increased the expression levels of MMPs (including MMP-1, −3, and −13) and decreased the expression of TIMP-1 in chondrocytes, consistent with previous reports [28, 29].

MMPs comprise a family of enzymes that facilitate extracellular matrix (ECM) turnover and breakdown. All members of the MMP family have been linked to disease development, notably arthritis, cancer metastasis, chronic inflammation, and neurological disorders [30]. Among all MMPs, MMP-13 has been reported to play a central role in the progression of OA due to its potent proteolytic effects on collagen II, the main component of the ECM. The specialized role of MMP-13 in bone development and disease progression has made it an attractive target for selective MMP-13 inhibitors as therapeutic compounds [31, 32]. MMP-1 is a secreted enzyme and has wide substrate specificity for degradation of collagens, aggrecan, versican, serpins, and tenascin-C [33]. The substrate specificity of MMP-3 is also broad; MMP-3 has been shown to break down a number of ECM proteins, including fibronectin, laminin, denatured collagens, and proteoglycans. In addition to ECM degradation, MMP-3 is involved in the activation cascades of MMP-13 and gelatinases. Since MMP-1 and MMP-3 play vital roles in ECM turnover, their regulation has been suggested to be useful in the treatment of OA [34]. Interestingly, our results demonstrated that the upregulated MMP-1, −3, and −13 expression in IL-1β-stimulated chondrocytes were all markedly decreased by biochanin A in a dose-dependent manner. In view of the effects of biochanin A on MMP-2 and MMP-9 as reported previously [10], we also detected the expresson and production of MMP-2 and MMP-9. However, the results were not statistically significant. TIMPs, the endogenous regulators of MMPs, play important roles in maintaining homoeostasis with MMPs. Imbalance between MMPs and TIMPs is a salient feature during OA progression, leading to disruption of the balance between ECM biosynthesis and degradation. Since the activities of MMPs can be inhibited by TIMPs, we investigated the effects of biochanin A on TIMP-1 expression; our results suggested upregulation of TIMP-1 by biochanin A in IL-1β–stimulated chondrocytes. Hence, we speculated that biochanin A may exert its chondroprotective effects by regulating the expression and activity of MMPs and TIMPs.

The NF-κB signaling pathway is known to play a vital role in OA [35, 36]. In the progression of OA, NF-κB transcriptional factors can be triggered by a number of stimuli, such as cytokines, excessive mechanical stress and degradation products of ECM. Activated NF-κB regulates several enzymes involved in matrix degradation, including MMP-1, −3, and −13 [37]. In general, NF-κB p65 is associated with IκB-α in an inactive form in the cytoplasm. Following IκB-α phosphorylation by stimuli, such as IL-1β, the nuclear localization signal is no longer masked, leading to translocation of NF-κB p65 to the nucleus [38]. Since biochanin A inhibited the NF-κB activation pathway in other experimental models [9, 39], we investigated the involvement of the NF-κB signaling pathway in MMP modulation by biochanin A in chondrocytes. In this study, we showed that biochanin A inhibited IκB-α degradation and then attenuated the activation of NF-κB p65. However, further studies of the mechanism underlying the effect of biochanin A in the treatment of OA are required.

In addition, we used the ACLT (anterior cruciate ligament transection) model to assess the effects of biochanin A *in vivo*, which has proven to be effective and reliable [40, 41]. Pickarski M et al. reported that matrix degradation and up-regulation of MMPs were obviously observed in the surgically-induced models of osteoarthritis [42]. Similar findings were reported by Li X et al., they found that MMP-13 was strikingly up-regulated in ACLT-induced OA [43]. Consistent with our *in vitro* data, the results of the *in vivo* study showed that the levels of MMP-1, −3, and −13 expression were decreased, while that of TIMP-1 was increased, in biochanin-A-treated cartilage compared with vehicle-treated controls. Moreover, we demonstrated that intra-articular injection of biochanin A ameliorated cartilage degradation during the progression of OA. Taken together, these findings confirm the potential value of biochanin A in the treatment of OA.

## Conclusions

In conclusion, we demonstrated at both the mRNA and protein levels that biochanin A inhibited the expression of MMP-1, −3, and −13 and increased the expression of TIMP-1, all of which are classic biomarkers of inflammation and cartilage degradation in OA. Our study provided strong evidence that these protective effects of biochanin A are mediated at least in part via inhibition of the NF-κB pathway. Taken together, these findings indicate that biochanin A exerts anticatabolic effects in the progression of OA and may reduce the rate of cartilage degradation. Therefore, biochanin A may be useful for the treatment and prevention of OA.

## Abbreviations

MMP: Matrix metalloproteinases
TIMP-1: Tissue inhibitor of metalloproteinases-1
IL-1β: Interleukin-1beta
OA: Osteoarthritis
NF-κB: Natural factor kappa B
IκB-α: Inhibitory kappa B-alpha
ACLT: Anterior cruciate ligament transection.
